# Supplementary material for: Neurons in the Nucleus papilio contribute to the control of eye movements during REM sleep
Source: Nat Commun. 2019 Nov 19;10:5225. doi: 10.1038/s41467-019-13217-y (PMC6864097; doi:10.1038/s41467-019-13217-y)
Supplement: Supplementary file 1 — Supplementary Information [file 41467_2019_13217_MOESM1_ESM.pdf]

Supplementary information:

# Neurons in the *Nucleus papilio* contribute to the control of eye movements during REM sleep.

Guttierez-Herrera, C., Girard, F. et al.

Supplementary figure 1

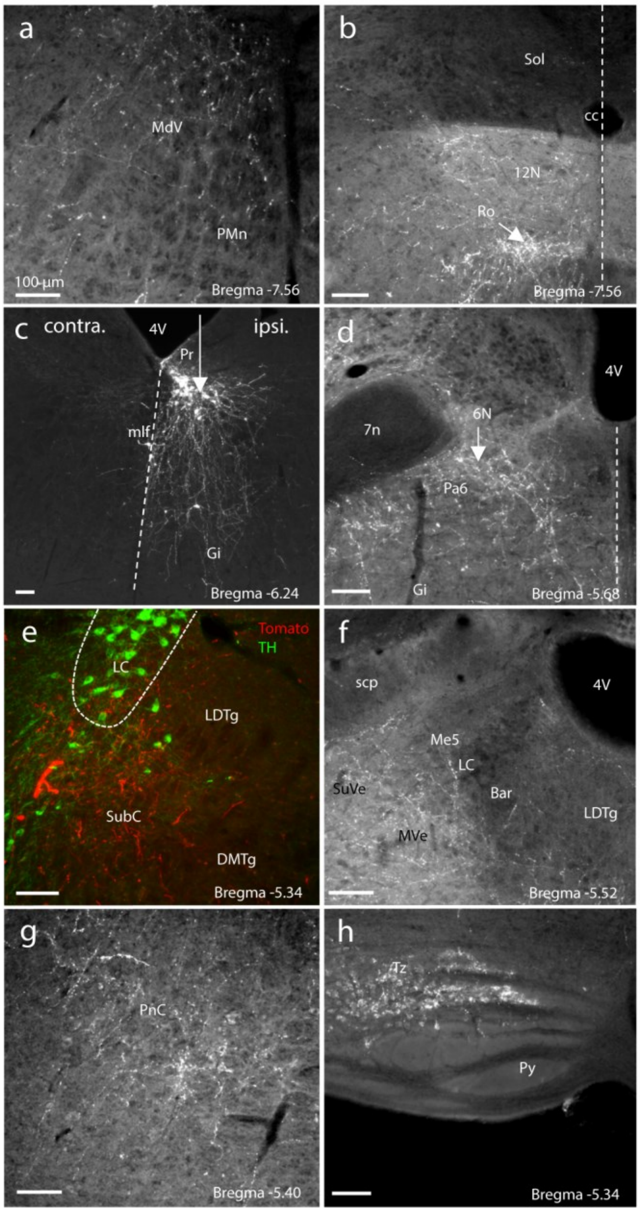


**Supplementary Fig.1: Anterograde tracing in *Calb1*::Cre mice.** The efferent connections of the *Nucleus papilio* reach both nuclei involved in eye movements and nuclei involved in REM sleep control. Representative micrographs of coronal sections at the indicated different brain levels (Bregma levels are indicated). Adenovirus injections were performed unilaterally in the $NP^{Calb}$, marked with a vertical arrow in (c). All other images show brain side contralateral to the injection site (dashed lines represent the midline). Strong terminal labelling was evident in the nucleus of the $6^{th}$

(d), as well as the nuclei of the $4^{th}$ and $3^{d}$ nerve (see main text and Fig.3b). The medullary reticular (a), Roller (b), vestibular (f), pontine (g), trapezoid (h) nuclei are rich in terminals derived from the $NP^{Calb}$. In panel (e), the section was stained for tyrosine hydroxylase, highlighting in green the *Locus coeruleus*. Notice a large number of axonal endings of the $NP^{Calb}$ in the subcoeruleus nucleus. Bar = 100 µm. Abbreviations: MdV: medullary reticular nucleus, ventral; PMn: paramedian reticular nucleus; Sol: nucleus of the solitary tract; cc: central canal; 12N: hypoglossal nucleus; RO: nucleus of Roller; Gi : gigantocellular reticular nucleus ; 4V: fourth ventricle; Pr : prepositus nucleus; mlf: medial longitudinal fasciculus; Gi : gigantocellular reticular nucleus ; 7n: facial nerve; 6N: abducens nucleus; Pa6: para-abducens nucleus; LC: *Locus coeruleus*; Bar: Barrington nucleus; SubC: subcoeruleus nucleus; LDTg: laterodorsal tegmental nucleus; DMTg: dorsomedial tegmental area; scp: superior cerebellar peduncle; SuVe: superior vestibular nucleus; MVe: medial vestibular nucleus; Me5: mesencephalic trigeminal nucleus; PnC: pontine reticular nucleus, caudal; py: pyramidal tract.

Supplementary figure 2

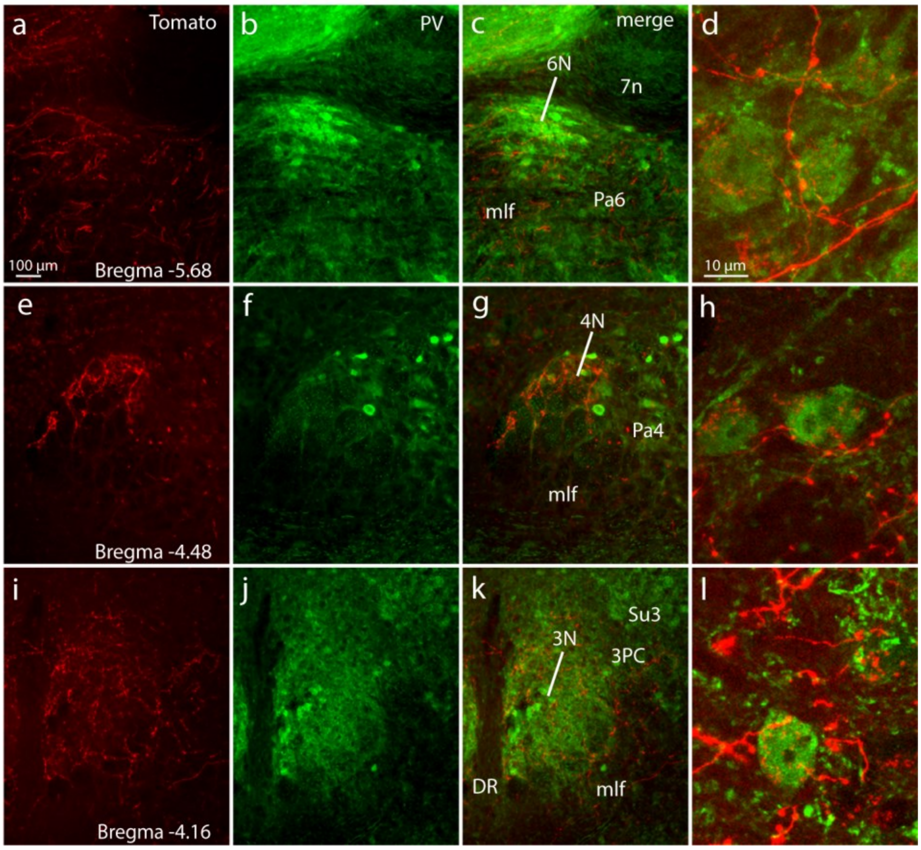


**Supplementary Fig. 2: NP$^{Calb}$ neurons send axons to the motoneurons of the three nuclei regulating EMs.** Parvalbumin-immunostaining (green, b, f, j) is used to reveal motoneuronal cell bodies within the abducens (6N, a-d), trochlear (4N, e-h) and oculomotor (3N, i-l) nuclei. All images correspond to coronal sections at the different Bregma levels indicated, from a *Calb1*::Cre mouse brain injected in the NP$^{Calb}$ with an AAV2/1.CAG.Flex.Tomato.WPRE.bGH (red). The rightmost panels (d, h, l) are confocal images showing Tomato-labelled terminals impinging on Parv-positive motoneurons within each of the nuclei.

Abbreviations: 6N: abducens nucleus; 7n: facial nerve; mlf: medial longitudinal fasciculus; Pa6: para-abducens nucleus; 4N: trochlear nucleus; Pa4: para-trochlear nucleus; 3N: oculomotor nucleus; Su3: supraoculomotor nucleus; 3PC: oculomotor nucleus, parvicellular; DR: dorsal raphe nucleus.

Supplementary figure 3

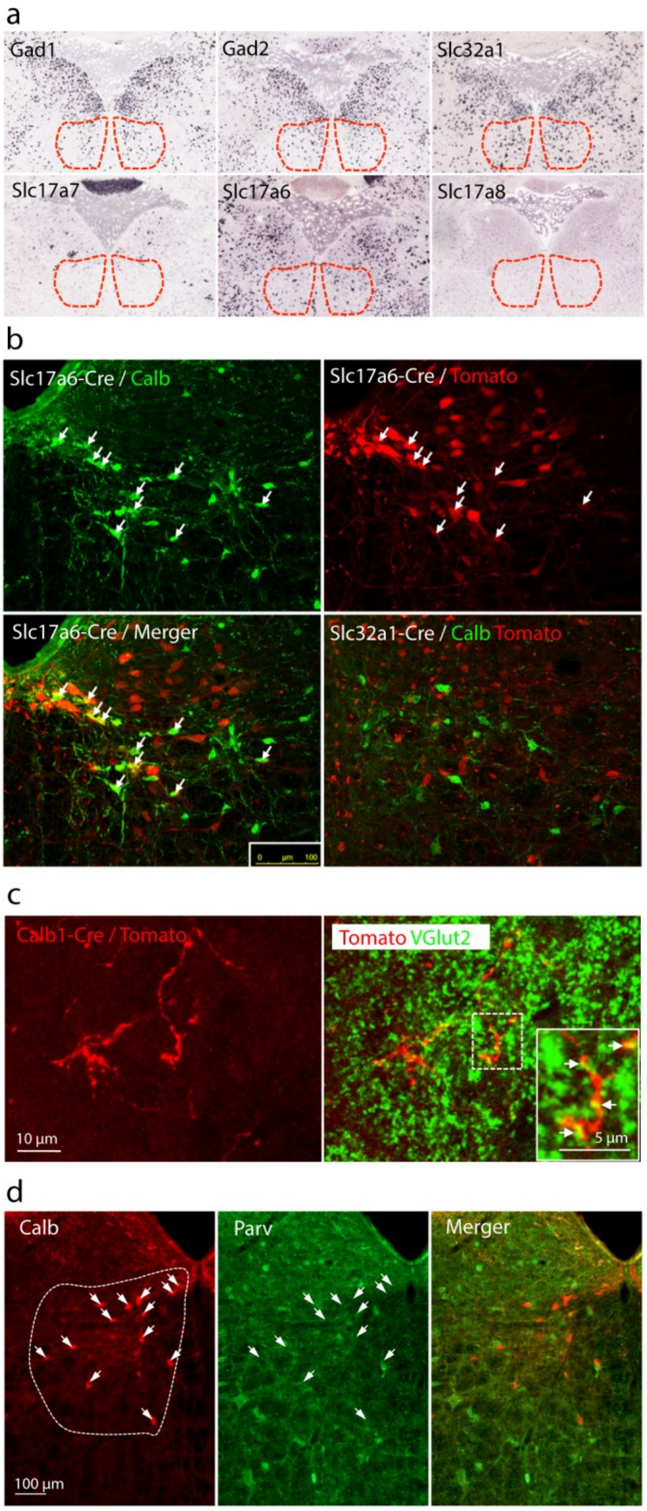


**Supplementary Fig. 3: Glutamatergic nature of some of the NP$^{Calb}$ neurons.** (a) In situ hybridization images taken from the Allen Brain Atlas (Image credit: Allen Institute; *Gad1*: http://mouse.brain-map.org/experiment/show/79556706; *Gad2*: http://mouse.brain-map.org/experiment/show/79591669, *Slc32a1*: http://mouse.brain-

map.org/experiment/show/72081554; *Slc17a6*: http://mouse.brain-map.org/experiment/show/73818754; *Slc17a7*: http://mouse.brain-map.org/experiment/show/75081210; *Slc17a8*: http://mouse.brain-map.org/experiment/show/71587918), highlighting the expression of the following genes in the NP$^{Calb}$ area: *Gad1* and *Gad2* (encoding glutamic acid decarboxylase GAD65/67), *Slc32a1* (encoding GABA/Glyt vesicular transporter), *Slc17a7*, *Slc17a6* and *Slc17a8* (encoding respectively vesicular glutamate transporters VGlut1, VGlut2 and VGlut3). The first three genes are hallmarks of GABAergic neurons, while the expression of one of the other three is the signature for glutamatergic neurons. The red dashed lines visualize the putative boundaries of the NP$^{Calb}$. (b) AAV2/1.CAG.Flex.Tomato.WPRE.bGH injected into the NP$^{Calb}$ in *Slc17a6*::Cre mice or *Slc32a1*::Cre mice was used to label respectively glutamatergic and GABAergic neurons. Immunostaining with anti-Calb antibody reveals Calb-immunoreactivity to be present in a significant proportion of Tomato-labelled *Slc17a6*-expressing neurons (33.8%, $n$ = 4 mice), showing their glutamatergic nature. No coexistence was observed between Calb-immunoreactive and GABAergic neurons (*Slc32a1*-Cre / Tomato). (c) VGlut2 immuno-staining investigated in Tomato-labelled terminals in the 3N (from a *Calb1*::Cre mouse injected with AAV-Tomato in the NP$^{Calb}$). The merged image (c, right) highlights the presence of VGlut2 immunoreactive dots along the Tomato-labelled terminals (indicated with white arrows in the inserted magnification). (d) NP$^{Calb}$ neurons (red, left, white arrows) do not co-express Parv (green, middle). A dashed line delineates the NP$^{Calb}$.

Supplementary figure 4

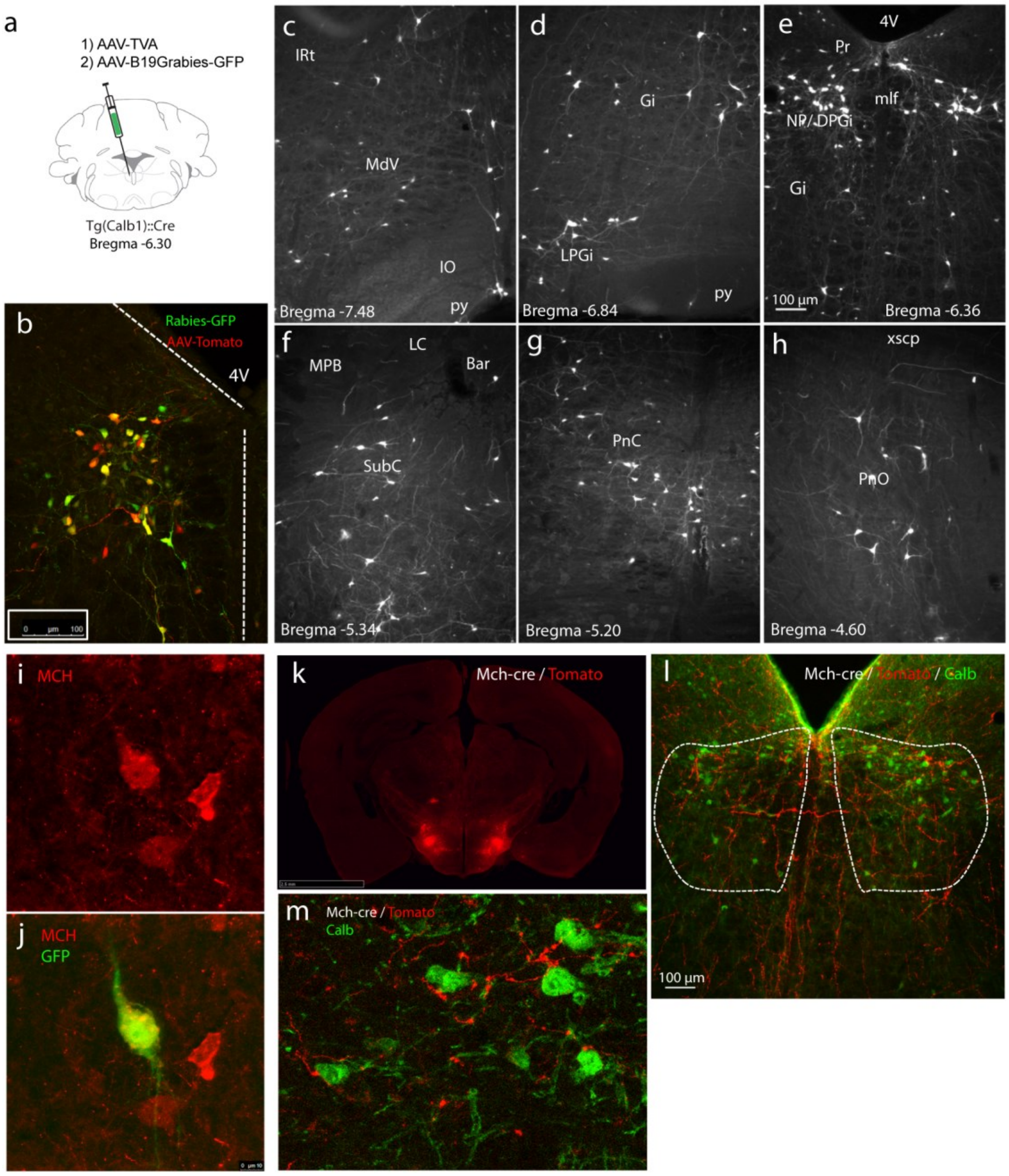


**Supplementary Fig. 4: Rabies-mediated trans-synaptic tracing in *Calb1*::Cre mice.** (a) Schematic representation of the stereotactic injections in *Calb1*::Cre mice of the adeno-associated viruses AAV-TVA and AAV-B19G, and of a modified Rabies-GFP. (b) The accuracy and efficiency of the injections was monitored by co-injection of AAV-Tomato in this *Calb1*::Cre mouse. (c-h) Images representing retrogradely GFP-stained neurons in various brain areas (see abbreviations for identification). Only

for panel (e) are both brain hemispheres shown, highlighting the injected NP$^{Calb}$. For the other images, only the right hemisphere is depicted. (i, j) A MCH-positive neuron (identified by immunostaining with anti-MCH antibody in red) within the lateral hypothalamus, that was retrogradely labelled with Rabies-GFP injected in the NP$^{Calb}$ of *Calb1*::Cre mouse. (k-m) AAV2/1.CAG.Flex.Tomato.WPRE.bGH, injected symmetrically in the lateral hypothalamus of an *Mch*::Cre mouse brain, targets MCH-expressing neurons (k), which are sending axons to the *medulla oblongata*, including the NP$^{Calb}$ neurons (l). A closer examination with confocal microscopy reveals some fluorescent terminals close to NP$^{Calb}$ neurons (m).

Abbreviations: IRt: intermedial reticular nucleus ; MdV: medullary reticular nucleus, ventral; IO: inferior olive; py: pyramidal tract; LPGi : lateral paragigantocellular nucleus ; Gi : gigantocellular reticular nucleus ; 4V: fourth ventricle; Pr : prepositus nucleus; DPGi: dorsal paragigantocellular nucleus; mlf : medial longitudinal fasciculus ; MPB: medial parabrachial nucleus; LC: *Locus coeruleus*; Bar: Barrington nucleus; SubC: subcoeruleus nucleus; PnC: pontine reticular nucleus, caudal; PnO: pontine reticular nucleus, oral; xscp: decussation of the superior cerebellar peduncle.

Supplementary figure 5

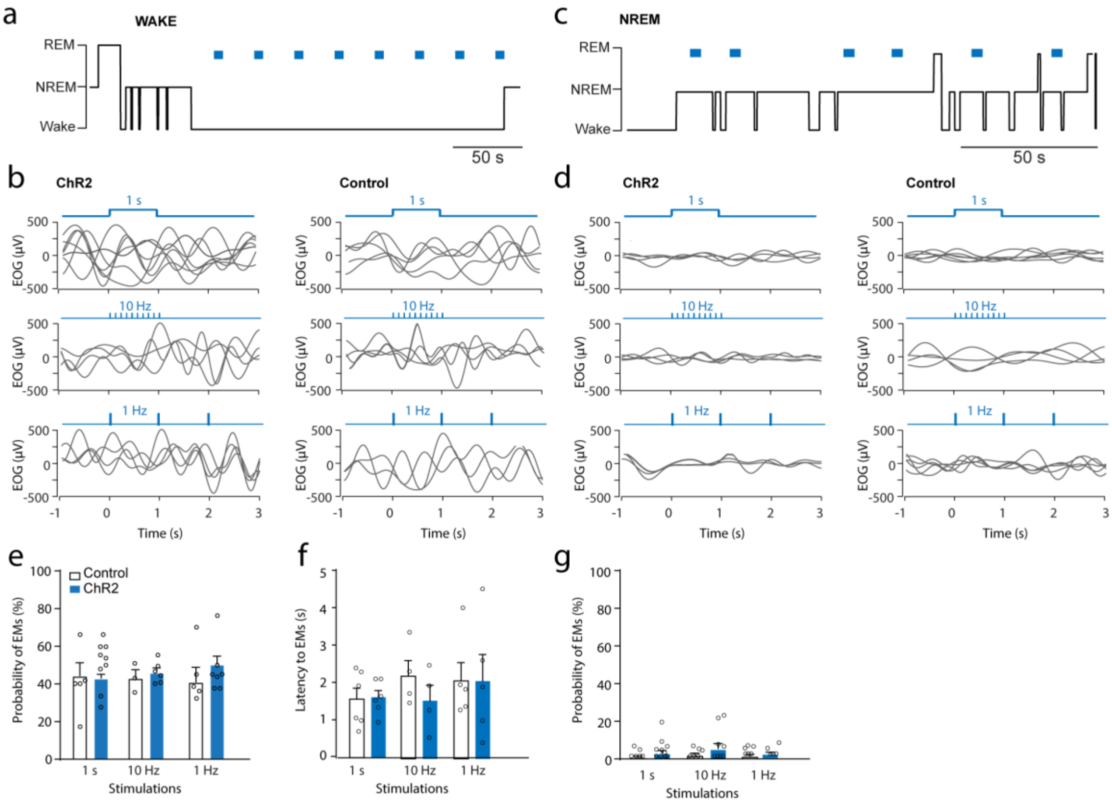


**Supplementary Fig. 5: EOG and EMs responses to optogenetic activation of NP$^{Calb}$ neurons during wake and NREM sleep.** (a, b) Representative hypnograms of optical activation of NP$^{Calb}$ neurons during wake (a) and NREM sleep (b). (c, d) EMs during wake (c) and NREM sleep (d) showing all detected averaged EMs per animal (grey lines) over-imposed and aligned to the onset of the light activation signals (blue line on the top) during the episode, 1 s before and 3 s after the onset of the laser. Traces show averaged EMs in response to the stimulation frequencies tested (1 s continuous light: *n* = 6; 10 Hz and 1 Hz: *n* = 4 mice) delineated by the blue traces on top of the graphs. (e) Summary data of the probability of an EM after the onset of the light activation during wakefulness in response to the stimulation frequencies tested for ChR2-transduced and control animals, respectively (for 1 s continuous light: *n* = 9 ChR2-transduced and 5 control animals, P

= 0.410, t = 1.439 respectively; For 10 Hz: *n* = 6 and 3, P = 0.977, t = 0.363; For 1 Hz: *n* = 6 and 5 mice, P = 0.997, t = 0.175; df = 28 for all conditions). (f) Summary data representing the latency from the onset of the light activation to the first EM during wakefulness in response to the stimulation frequencies tested for ChR2 and control animals (1 s continuous light: *n* = 6, P = <0.99, t = 0.010; 10 Hz: *n* = 4, P = 0.726, t = 0.951; and 1 Hz: *n* = 5 mice, P = <0.99, t= 0.087; df = 24 for all conditions tested). (g) Summary data of the probability of an EM after the onset of the light activation during NREM sleep in response to the stimulation frequencies tested for ChR2 and controls (1 s continuous light: *n* = 6 and 10, P = 0.0578, t = 0.178; 10 Hz: *n* = 8 and 6; and 1 Hz: *n* = 6 and 5 mice, P = 0.953, t = 0.471; df = 35 respectively). *: P<0.001 significance 2-way ANOVA followed by Sidak *post hoc* test for multiple comparisons. Circles represent the average per animal of all the animals included in the analysis. Error bars represent S.E.M values.

# Supplementary figure 6

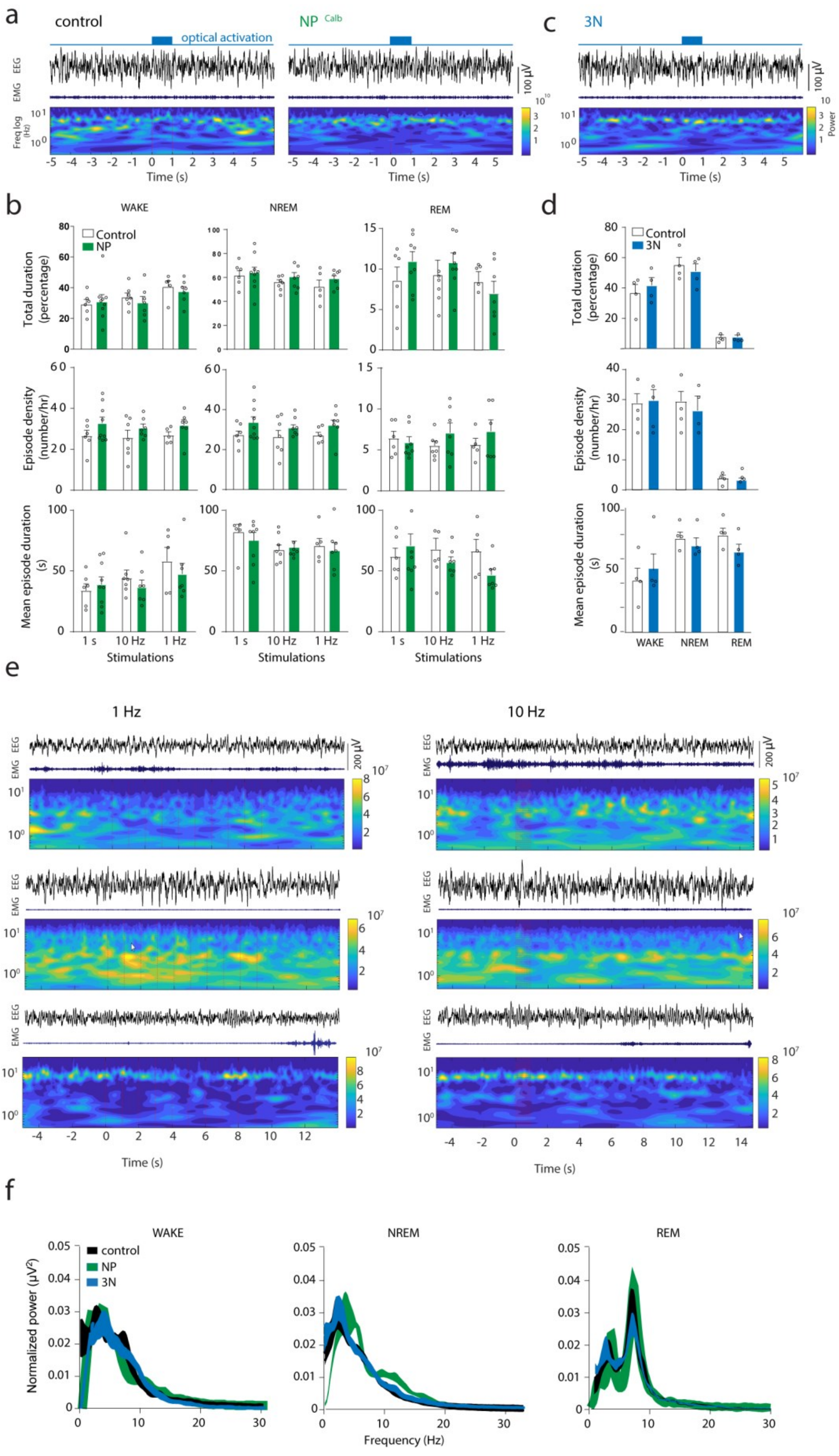

**Supplementary Fig. 6: Wake-Sleep architecture and power spectrum in response to optogenetic activation of $NP^{Calb}$ neurons.** (a, c) Representative traces in one animal of brain cortical activity (EEG) and muscle activity (EMG) in response to light activation of $NP^{Calb}$ neurons during REM sleep in control animals (a, left) and ChR2-transduced ones (a, right), and in response to light activation of $NP^{Calb}$ neuron terminals in the 3N (c). In each of the cases, bottom represents time frequency analysis of the representative EEG trace shown on the top. (b) Wake-sleep analysis during light semi-chronic stimulation at 1Hz, 10 Hz and 1 s continuous light for ChR2-transduced and control animals (1 s: $n$ = 9 and 6 mice; 10 Hz: $n$ = 7 and 7; 1 Hz: $n$ = 7 and 5, respectively) in the $NP^{Calb}$ soma. Shown are duration of wake (left), NREM (middle) and REM sleep (right) expressed in percentage (%) per hour (*top*) (for wake, NREM and REM p = 0.139, 0.08, and 0.46 and F(2.,35) = 2.08, 2.58, and 0.788 respectively), episode density (number of episodes per hour, middle) (for wake, NREM and REM p = 0.841, 0.791, and 0.96 and F(2,35) = 0.168, 0.23, and 0.04 respectively) and mean episode duration (bottom) (for wake, NREM and REM p = 0.093, 0.193, and 0.489 and F(2,35) = 2.54, 1.727, and 0.728 respectively). Values represent the average per animal (circles) of triplicate stimulation sessions. (d) Results from light activation of ChR2 terminals derived from the $NP^{Calb}$ neurons in 3N (P = 0.356 and F (2,18) = 1 for % of sleep; P = 0.30.971 and F = 0.001 for episode density; P = 0.644 and F = 0.221 for mean bout duration; measurements were carried out in duplicates). Error bars represent S.E.M values. Statistics were carried out using 2-way ANOVA, Sidak post hoc test for multiple comparisons. (e) Time frequency analysis during wake (top), NREM (middle) and REM-sleep (bottom) in response to $NP^{Calb}$ neuron activation using 1 Hz (left) or 10 Hz light pulses (right). Frequencies are represented in logarithmic scale (*/lnlog).* (f) Power

spectrum distribution of control animals (black), $NP^{Calb}$ cell soma activation (green) and 3N terminal activation (blue), during wake, NREM and REM sleep.

## Supplementary figure 7

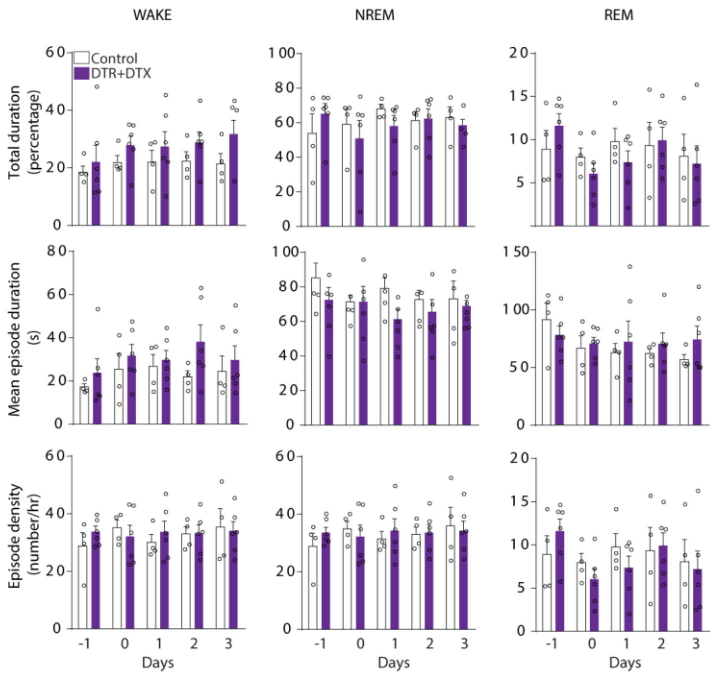


**Supplementary Fig. 7: Wake-Sleep architecture after genetic ablation of NP$^{Calb}$ neurons.** (Top): Duration of wake, NREM and REM sleep expressed in percentage (%), during a period of 1 hour at the same circadian time to the activation experiments, subsequent to saline injection (Day -1), DTX injection (Day 0) and three consecutive days post-injection of the toxin (Day 1 to Day 3). ($P = 0.696$, $F_{(4,32)} = 0.556$ for wake; $P = 0.815$, $F = 0.388$ for NREM; and $P = 0.231$, $F = 1.481$ for REM). (Middle): Mean episode duration (wake: $P = 0.676$, $F_{(4,32)} = 0.584$; NREM: $P= 0.672$, $F_{(4,32)} = 0.591$; REM bouts: $P= 0.336$, $F_{(4,32)} = 0.186$). (Bottom): Number of wake, NREM and REM bouts per hour (episode density; $P = 0.904$, $F_{(4, 32)} = 0.256$ for wake; $P = 0.903$, $F = 0.257$ for NREM; $P = 0.359$, $F = 1.124$ for REM). *n* = 4 and 6, control and DTR-mice, respectively. We found no statistical significance in these sleep parameters across days using 2-way ANOVA, Sidak post hoc test for multiple comparisons. Error bars represent S.E.M values.
