# Supplementary material for: Neurons in the Nucleus papilio contribute to the control of eye movements during REM sleep
Source: Nat Commun. 2019 Nov 19;10:5225. doi: 10.1038/s41467-019-13217-y (PMC6864097; doi:10.1038/s41467-019-13217-y)
Supplement: Supplementary file 3 — Reporting Summary [file 41467_2019_13217_MOESM3_ESM.pdf]

nature research

Corresponding author(s): Marco R Celio

Last updated by author(s): Oct 16, 2019

Reporting Summary

Nature Research wishes to improve the reproducibility of the work that we publish. This form provides structure for consistency and transparency in reporting. For further information on Nature Research policies, see Authors & Referees and the Editorial Policy Checklist.

Statistics

For all statistical analyses, confirm that the following items are present in the figure legend, table legend, main text, or Methods section.

n/a	Confirmed	
☐	☒	The exact sample size (*n*) for each experimental group/condition, given as a discrete number and unit of measurement
☐	☒	A statement on whether measurements were taken from distinct samples or whether the same sample was measured repeatedly
☐	☒	The statistical test(s) used AND whether they are one- or two-sided<br>*Only common tests should be described solely by name; describe more complex techniques in the Methods section.*
☐	☒	A description of all covariates tested
☐	☒	A description of any assumptions or corrections, such as tests of normality and adjustment for multiple comparisons
☐	☒	A full description of the statistical parameters including central tendency (e.g. means) or other basic estimates (e.g. regression coefficient) AND variation (e.g. standard deviation) or associated estimates of uncertainty (e.g. confidence intervals)
☐	☒	For null hypothesis testing, the test statistic (e.g. *F*, *t*, *r*) with confidence intervals, effect sizes, degrees of freedom and *P* value noted<br>*Give P values as exact values whenever suitable.*
☒	☐	For Bayesian analysis, information on the choice of priors and Markov chain Monte Carlo settings
☒	☐	For hierarchical and complex designs, identification of the appropriate level for tests and full reporting of outcomes
☒	☐	Estimates of effect sizes (e.g. Cohen's *d*, Pearson's *r*), indicating how they were calculated

Our web collection on statistics for biologists contains articles on many of the points above.

Software and code

Policy information about availability of computer code

Data collection	Data collection was performed using AM system coupled to a signal aquisition algorithms written in Mathworks Matlab (v. 2015b) form . Algorithms are not published, but will be made available to readers and reviewers on request.
Data analysis	Signal processing was performed using custom written algorithms in Mathworks Matlab (v. 2015b). EOG signals were detected and cored using spike 2 sofware. Sleep quantifications were done using algorithms that are not published, but will be made available to readers and reviewers on request. Statistical analysis was performed using preset algorithms in Graphpad Prism (v. 7)

For manuscripts utilizing custom algorithms or software that are central to the research but not yet described in published literature, software must be made available to editors/reviewers. We strongly encourage code deposition in a community repository (e.g. GitHub). See the Nature Research guidelines for submitting code & software for further information.

Data

Policy information about availability of data

All manuscripts must include a data availability statement. This statement should provide the following information, where applicable:

- Accession codes, unique identifiers, or web links for publicly available datasets
- A list of figures that have associated raw data
- A description of any restrictions on data availability

The authors declare that the data supporting the findings of this study are available within the paper and its supplementary information files.

Field-specific reporting

Please select the one below that is the best fit for your research. If you are not sure, read the appropriate sections before making your selection.

☒ Life sciences ☐ Behavioural & social sciences ☐ Ecological, evolutionary & environmental sciences

For a reference copy of the document with all sections, see nature.com/documents/nr-reporting-summary-flat.pdf

Life sciences study design

All studies must disclose on these points even when the disclosure is negative.

Sample size	No statistical tests were used to determine sample sizes. Instead, animal groups sizes were determined based on published literature from our group (see: Jego et al., 2013 Nat. Neuroscience; Herrera et al., 2016 Nat. Neuroscience).
Data exclusions	Animals with aberrant circadian distribution of sleep-wake cycle (<10 %) were discarded from this study. Only animals with EOG, EEG and EMG signals were taken into consideration for the study. Animals with no viral expression were discarded from optogenentic expreriments. For the probability, latency, and Gaussian distribution analysis, animals with less than two REMs episodes in the hour of optogenetic stimulation or with poor EOG signals were not included in the analysis.
Replication	Optogenetic stimulation expreriments were done in at least duplicates. State specific optogenetic experiments were performed on at least 10 episodes per sleep state. We considered at least 2 different cohorts of animals.
Randomization	Sleep experiments animals were recorded in pairs, one control and one experimental animals, where the experimenter was blinded to the animal condition. Optogenetic stimulations were performed in the same fashion in both control and experimental animals.
Blinding	Experimenters were bind to the condition of the animals untill the data analysis were performed. For sleep scoring, 2 independent experimenters performed the scoring.

Reporting for specific materials, systems and methods

We require information from authors about some types of materials, experimental systems and methods used in many studies. Here, indicate whether each material, system or method listed is relevant to your study. If you are not sure if a list item applies to your research, read the appropriate section before selecting a response.

Materials & experimental systems

n/a	Involved in the study	
☐	☒	Antibodies
☒	☐	Eukaryotic cell lines
☒	☐	Palaeontology
☐	☒	Animals and other organisms
☒	☐	Human research participants
☒	☐	Clinical data

Methods

n/a	Involved in the study	
☒	☐	ChIP-seq
☒	☐	Flow cytometry
☒	☐	MRI-based neuroimaging

Antibodies

Antibodies used	mouse and rabbit anti-Calbindin-D28k (Swant, Marly, Switzerland), mouse and rabbit anti-c-fos (respectively Abcam, Cambridge, UK and Merck, Darmstadt, Germany), rabbit anti-GFP (Life Technologies Europe, Zug, Switzerland), rabbit anti-MCH (Phoenix Pharmaceuticals, Karlsruhe, Germany), mouse and rabbit anti-parvalbumin (Swant, Marly, Switzerland), mouse anti-tyrosine hydroxylase (Immunostar, Houston, USA), rabbit anti-VGlut2 (Synaptic Systems, Goettingen, Germany).
Validation	All antibodis are commercially-available, and were used according to manufacturer's instructions

Animals and other organisms

Policy information about studies involving animals; ARRIVE guidelines recommended for reporting animal research

Laboratory animals	The following mouse lines were obtained from Jackson laboratory:<br>B6.Cg-Calb1tm1.1(folA/Cre)/Hze/J<br>Tg(Pmch-cre)1Lowl<br>Slc17a7tm1.1(Cre)/Hze/J<br>Slc17a6tm2(Cre) lowl/J<br>Slc32a1tm2(Cre) lowl/J

Other animals were C57BL/6J mice (from our animal facility) of both genders , and 3 months old Wistar rats (Janvier, Lyon, France) (n=3 males and 3 females).

For optogenetic experiments, only male B6.Cg-Calb1tm1.1(folA/Cre)/Hze/J were used.
Viral injections were performed at 6-10 weeks of age. Instrumentation and recordings were perfomed at 10-16 weeks of age.

Wild animals	N/A
Field-collected samples	N/A
Ethics oversight	The study was approved by the Committee for Animal Experimentation of the Cantons of Fribourg (2015_FR18), respectively Bern (2015_FR18+) (Switzerland).

Note that full information on the approval of the study protocol must also be provided in the manuscript.
